# A comprehensive, multiscale framework for evaluation of arrhythmias arising from cell therapy in the whole post-myocardial infarcted heart

**DOI:** 10.1038/s41598-019-45684-0

**Published:** 2019-06-25

**Authors:** Joseph K. Yu, William Franceschi, Qinwen Huang, Farhad Pashakhanloo, Patrick M. Boyle, Natalia A. Trayanova

**Affiliations:** Institute for Computational Medicine, Johns Hopkins University, 3400 N Charles St, 216 Hackerman Hall, Baltimore, Maryland 21218 USA

**Keywords:** Cardiac regeneration, Computational models

## Abstract

Direct remuscularization approaches to cell-based heart repair seek to restore ventricular contractility following myocardial infarction (MI) by introducing new cardiomyocytes (CMs) to replace lost or injured ones. However, despite promising improvements in cardiac function, high incidences of ventricular arrhythmias have been observed in animal models of MI injected with pluripotent stem cell-derived cardiomyocytes (PSC-CMs). The mechanisms of arrhythmogenesis remain unclear. Here, we present a comprehensive framework for computational modeling of direct remuscularization approaches to cell therapy. Our multiscale 3D whole-heart modeling framework integrates realistic representations of cell delivery and transdifferentiation therapy modalities as well as representation of spatial distributions of engrafted cells, enabling simulation of clinical therapy and the prediction of emergent electrophysiological behavior and arrhythmogenensis. We employ this framework to explore how varying parameters of cell delivery and transdifferentiation could result in three mechanisms of arrhythmogenesis: focal ectopy, heart block, and reentry.

## Introduction

Ischemic heart disease remains the leading cause of death in the United States and globally^1,2^. In its most severe presentation, acute plaque rupture can occur that restricts blood flow to the myocardium causing myocardial infarction (MI), or cardiac muscle damage. The inability to regenerate lost or injured cardiomyocytes (CMs) is an inherent biological limitation of the adult human heart that remains unaddressed by current clinical treatments. Subsequently, chronic cardiac overload and progressive myocardial remodeling can give rise to congestive heart failure (HF) and the need for heart transplantation^3^. Cell-based heart repair seeks to address the onset of HF by halting remodeling and restoring ventricular contractility to the post-MI heart.

Depending on the type of cells delivered, potential therapeutic mechanisms can range from non-myogenic paracrine effects^4–9^, indirect remuscularization^10–13^, to direct remuscularization^14–25^. Various types of cells have been tested in preclinical and clinical studies and include skeletal myoblasts^26–28^, bone marrow-derived stem cells^6,29,30^, endothelial progenitor cells^31–33^, mesenchymal stem cells^5,34^, various endogenous cardiac stem cells^8,10^, pluripotent stem cell-derived cardiac progenitor cells^17,35^, pluripotent stem cell-derived CMs (PSC-CMs)^16,18,21,23,24^, and transdifferentiated induced CMs (iCMs)^36,37^. Cell types that do not differentiate into myocytes (bone marrow-derived stem cells, endothelial progenitor cells, and some endogenous cardiac stem cells) are believed to act through non-myogenic paracrine and indirect remuscularization mechanisms; specifically, they secrete proangiogenic and antiapoptotic factors, aid in reverse remodeling, or induce the proliferation of native ventricular CMs. In contrast, the introduction of *de novo* CMs either through PSC-CMs, iCMs, or cardiac progenitor cells that subsequently differentiate are believed to primarily act through direct remuscularization mechanisms by replacing lost CMs with new ones in the post-MI ventricles.

Despite promising results indicating cardiac functional improvement, ventricular tachycardia (VT) has been observed in studies implementing direct remuscularization strategies, highlighting the need to better understand how cells integrate electromechanically into the myocardium. Postoperative VTs were observed in several clinical pilot studies of skeletal myoblast in post-MI heart failure patients^38–40^. In the MAGIC trial^28^, incidences of VT were two times greater in patients treated with skeletal myoblasts compared to placebo; VTs also tended to occur during the early postoperative period in treated patients. These postoperative VTs could be attributed to the inability of skeletal myoblasts to express connexin-43 (Cx43), electrically couple, and beat in sync with the myocardium^41–43^. To address this limitation, alternative approaches have focused primarily on cardiac progenitor cells, PSC-CMs, and iCMs as sources for *de novo* CMs capable of electromechanical integration. Despite no instances of arrhythmia reported in early small-animal studies^22,23^, severe acute ventricular arrhythmias were observed following PSC-CM injections (>1 × 10^8^ PSC-CMs) into infarcted nonhuman primate hearts^24^. The exact mechanisms of VT remain primarily speculative. Further highlighting the need to understand the electromechanical coupling of *de novo* CMs, PSC-CM engineered into cell sheets have also been observed to exhibit arrhythmic activity^44^. Mechanistic investigations into arrhythmogenesis arising from cell-based heart repair is extremely challenging due to the difficulty of controlling many *in vivo* experimental variables related to the therapy (i.e., PSC-CM differentiation purity, cell migration and engraftment, infarct size and geometry). Multi-scale, biophysical-detailed cardiac electrophysiology modeling can however aid in this endeavor.

Cardiac electrophysiology modeling has become a vital tool for mechanistic enquiries across various scales of structural hierarchy, providing insight that cannot be acquired clinically or experimentally^45–47^. Developing the ability to simulate cell therapy in a realistic manner within a biophysically-detailed, whole-heart modeling infrastructure is an important step in the quest to understand the mechanisms responsible for the unwanted emergence of arrhythmias. Identifying the primary drivers of ventricular arrhythmia and subsequently devising strategies to eliminate them are important next steps towards successful clinical translation of cell-based heart repair with *de novo* CMs. Predictive modeling of heart repair with *de novo* CMs has a great potential to complement and guide *in vivo* large-animal studies by providing an accurate and efficient platform for understanding the dynamics of arrhythmogenesis and assessing the feasibility of strategies for mitigating it.

The objective of this study is to develop and utilize a novel, comprehensive framework for simulating the electrophysiological consequences arising from heart repair with *de novo* CMs. To achieve this, we represent and integrate key properties of therapy modality, cell engraftment, and myofibril alignment within a whole-heart modeling platform, ensuring accuracy and broad applicability of the simulation framework. We then demonstrate the utility of the framework with a set of diverse illustrative examples that identify potential mechanisms of ventricular arrhythmias – focal ectopy, heart block, and reentry –arising under different circumstances in the repaired heart. To highlight the flexibility and modularity of our framework, we conduct simulation studies with both human and animal (rabbit) heart models.

Our methodology enables the identification of key therapeutic parameters that mitigate arrhythmogenesis, which could guide clinical best practices and inform regulatory policy in cardiac regenerative therapies in the near future. Combined with patient-specific, clinical image-based heart modeling^47–49^, our simulation framework enables clinical feasibility studies in stratifying patient arrhythmia risk and treatment optimization. The approach presented here makes an important leap forward in cardiac regenerative therapy by integrating predictive computational cardiac electrophysiology modeling with cell-based heart repair and tissue engineering.

## Results

### Overview of key components of the cell therapy simulation framework at each scale of structural hierarchy

**Figure 1:**
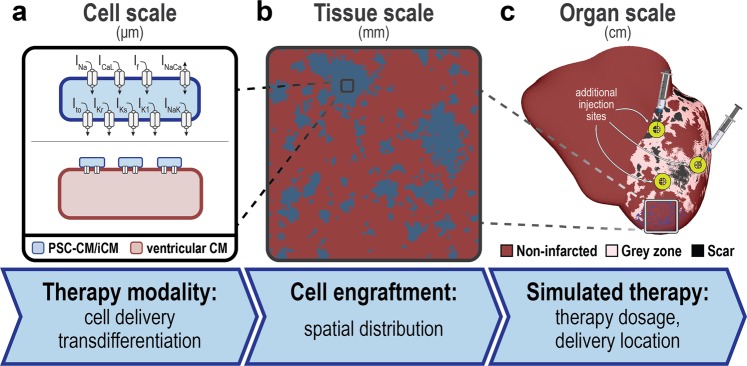
Hierarchy of the multi-scale whole-heart framework for simulating the electrophysiological effects of cell-based heart repair with *de novo* cardiomyocytes. (**a**) At the cell scale, two therapy modalities are represented: cell delivery (top) or transdifferentiation (bottom). (**b**) At the tissue scale, heterogeneous spatial distributions of remuscularized tissue (blue) among the native myocardium (red) is modeled. (**c**) At the organ scale, localized delivery is simulated into individualized 3D heart models of post-MI patients and the emergence of arrhythmia can be observed. Models are reconstructed from contrast-enhanced clinical MRIs and represent the distributions of grey zone (i.e., peri-infarct) and scar tissue.

Key properties of heart repair with *de novo* CMs were incorporated at the appropriate scale in a modular, multiscale cardiac electrophysiology simulation framework that leverages image-based, three-dimensional (3D) heart modeling. At the cell scale (Fig. 1a), representations of two primary therapy modalities were incorporated: cell delivery or transdifferentiation. At the tissue scale (Fig. 1b), realistic spatial distributions of engrafted *de novo* CMs were represented. At the organ scale (Fig. 1c), we simulate dose and site-specific effects of localized treatment in a 3D whole heart model of post-MI, allowing us to study emergent behaviors and to investigate the effects of delivery parameters on arrhythmogenesis. Features of this novel simulation framework are described in detail in the following sections.

### Cell scale modeling: therapy modality

Advances in experimental techniques and a broader understanding of cardiac development^50^ have enabled the generation of functional *de novo* CMs. *De novo* CMs can be introduced into the heart either through the cell delivery of exogenous PSC-CMs or the transdifferentiation of non-myocytes into iCMs; we do not simulate the introduction of cardiac progenitor cells because very few of the surviving cells differentiate into *de novo* CMs^51^. While adult ventricular CMs have a stable, diastolic membrane potential (−90 mV) and only depolarize when stimulated, PSC-CMs and iCMs exhibit a more fetal-like phenotype arising from differences in cell morphology and ion channel expression^52^; specifically, they have a more depolarized diastolic membrane potential, lower action potential (AP) amplitude, slower upstroke velocity, and exhibit intrinsic automaticity^53^. Because of these broad similarities and a lack of detailed electrophysiological characterization of iCMs, we represent PSC-CM and iCM membrane kinetics with the Paci *et al*. ionic model^54^ for a generic ventricular-like human-induced PSC-CM; we however alter its cell scale representation, as described below, to better simulate the differences arising from PSC-CM and iCM integration among native myofibers due to therapy. The ventricular-like subtype was selected because it is the most appropriate cardiac subtype for cardiac repair. New PSC-CM or iCM ionic models can be easily incorporated as they are developed due to the modularity of our simulation framework.

**Figure 2:**
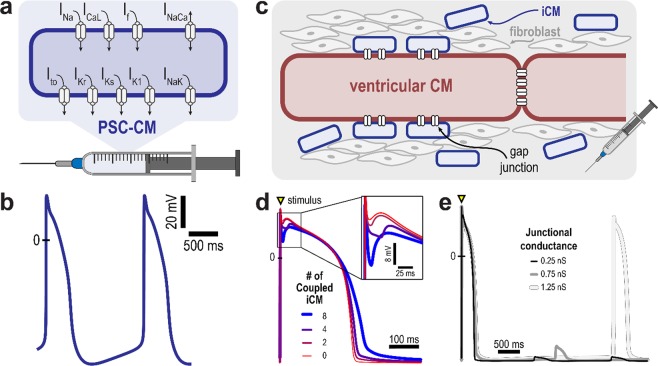
Cell scale therapy modalities and resulting action potentials. (**a**) In cell delivery, remuscularized regions are represented as comprised of PSC-CMs only; shown are ionic currents of the PSC-CM membrane model. **(b)** Simulated PSC-CMs action potentials illustrate intrinsic automaticity arising from immaturity; a beat is generated every 1.6 s. (**c**) Induced cardiomyocytes (iCMs) arising from the transdifferentiation of fibroblasts electrically couple and exert an electrotonic effect on an adjacent, host ventricular CM; remuscularized regions are represented as comprised of a ventricular CM resistively coupled to multiple iCMs. The electrotonic effect of iCMs on a ventricular CM action potential is modulated by (**d**) the number of iCMs and (**e**) the junctional conductance. (**d**) Increasing the number of coupled iCMs from 0 to 8 results in prolongation of the ventricular CM action potential duration and increase in the phase I repolarization notch (inset); junctional conductance is 0.75 nS. (**e**) Spontaneous action potentials are observed in a ventricular CM coupled to 4 iCMs with junctional conductance of 1.25 nS. Transient subthreshold depolarizations occur at lower values of junctional conductance.

Cell delivery via intramyocardial injection or transplantation of engineered cell sheets gives rise to contiguous regions of engrafted PSC-CMs^18,21,23,24^. Consequently, remuscularized regions with engrafted PSC-CM in the framework are assumed to only consist of cells with PSC-CM membrane kinetics (Fig. 2a). The PSC-CM AP exhibits a maximum diastolic potential of around −76 mV and intrinsic spontaneous rate of 37.5 bpm (Fig. 2b).

Contrastingly, transdifferentiation gives rise to iCMs in the cardiac interstitium around existing, native ventricular CMs and myofibers^36,37^. The reason for this is that transdifferentiation is initiated by viral vectors^36,55^ or microRNAs^56^ targeting fibroblasts surrounding the infarct^36,55–58^. Histological section of post-MI, murine hearts treated via transdifferentiation have indicated patchy distribution of iCMs among existing ventricular CMs; large regions of contiguous iCMs in border zone or scarred myocardium have not been reported. Thus, remuscularized regions of engrafted iCMs are modeled as a number of iCMs resistively coupled to a host adult ventricular CM (Fig. 2c). Figure 2d,e present the electrotonic effect of iCMs on a host ventricular CM action potential. Increasing the number of resistively coupled iCMs (from 2 to 8, each with junctional conductance of 0.75 nS) increased the phase I repolarization notch and prolonged the action potential duration of the host ventricular CM (Fig. 2d). Illustrated in Fig. 2e is a cell-level, arrhythmogenic effect of transdifferentiation arising from iCM automaticity: for certain values of junctional conductance (above 1.00 nS), a spontaneous action potential is elicited in the host ventricular CM; below these conductance values, only subthreshold depolarizations were observed.

### Tissue scale modeling: spatial distribution of *de novo* CMs

Localized cell delivery and transdifferentiation *in vivo* can result in noncontiguous clusters of remuscularized regions comprised of engrafted *de novo* CMs due to a combination of cell death, cell migration, and clonal expansion^21,24,36,59,60^. Our framework modelled this heterogeneous distribution of engrafted *de novo* CMs around sites of intramyocardial injection. A similar methodology can be applied to model *de novo* CM engraftment achieved via systemic or intracoronary delivery, a significantly less invasive delivery methodology but one that requires additional development to achieve notable engraftment.

**Figure 3:**
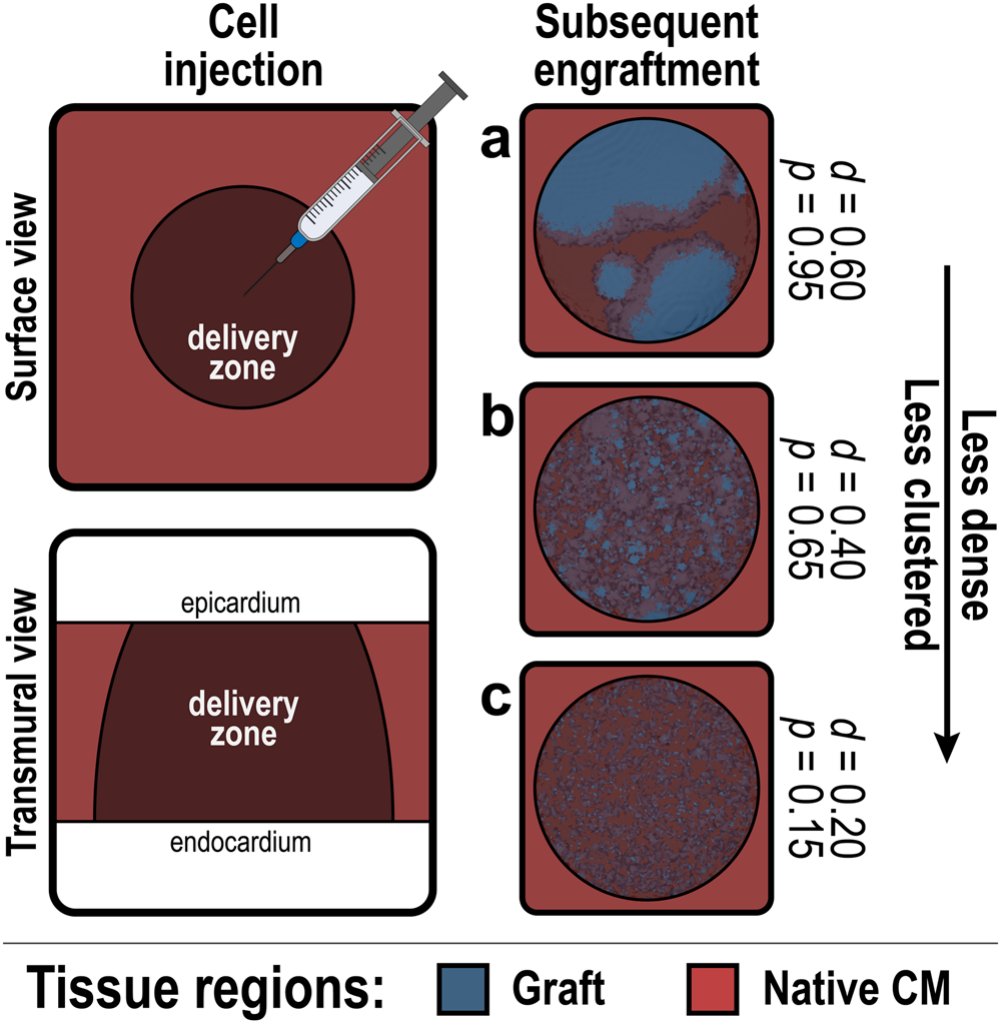
Tissue scale spatial distribution of cell engraftment following cell delivery or transdifferentiation. Stochastic spatial distributions of remuscularized tissue grafts (blue) arising from cell delivery or transdifferentiation are generated within a prescribed delivery zone (dark red) around the site of injection. (**a**–**c**) Generated clusters of engrafted cells for three different combinations of *d* (density) and *p* (clustering).

Realistic spatial distributions of engrafted *de novo* CMs (Fig. 3) were generated by adapting a previously described algorithm^46,61^. Within the 3D delivery zone around an injection site where *de novo* CMs engraft, stochastic spatial distributions of engrafted cells were generated based on two parameters, *d* and *p*; these parameters modulated the density or cell dosage (by volume fraction of delivery zone) and degree of spatial clusteredness, respectively. Different parameter combinations resulted in noticeably different *de novo* CM spatial distributions (Fig. 3a–c) consistent with experimentally observed patterns of cell engraftment^62,63^. Variability in host-graft coupling observed in the post-MI heart following cell delivery^22,23^ can be incorporated by adjusting tissue conductivity within individual PSC-CM clusters. Additional details can be found in the Methods section.

### Organ scale modeling: simulating localized delivery into a 3D whole-heart model

Contemporary whole-heart models incorporate 3D individualized heart geometry, tissue composition, and myofibril structure obtained from various imaging modalities^48,64–66^. Our group has pioneered the development of individualized heart models that incorporate distribution of structural remodeling from clinical late gadolinium enhanced (LGE)-MRI for understanding mechanisms of arrhythmia^49,65,67,68^, arrhythmia risk stratification^48^, and the development of personalized treatment planning^48,69^. In the case of post-MI, regions of non-infarcted, grey zone (i.e., peri-infarct border zone), and scar in the heart model are determined by MRI signal intensity^48,70^. Additional structural components such as the Purkinje system, the network of specialized conducting fibers responsible for fast excitation of the ventricles, has also been included in heart models^71,72^. These advances in whole-heart model construction lay the foundation for the current platform which enables us to simulate cell therapy at the level of the whole heart.

**Figure 4:**
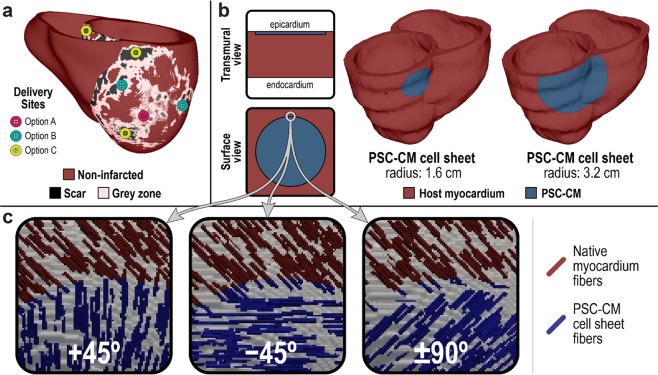
Organ scale transplantation of PSC-CMs into a personalized 3D heart model of ischemic cardiomyopathy. (**a**) Any combination of delivery locations and cell dosages can be simulated in an individualized, 3D whole heart model; PSC-CMs can be distributed across multiple sites around the scar, grey zone, and non-infarcted tissue. (**b**) In the context of PSC-CM cell sheets, cell dosage can be adjusted by altering sheet size or thickness. (**c**) Myofibril discontinuity arising from the misorientation of cell sheets with aligned PSC-CMs can also be simulated.

**Figure 5:**
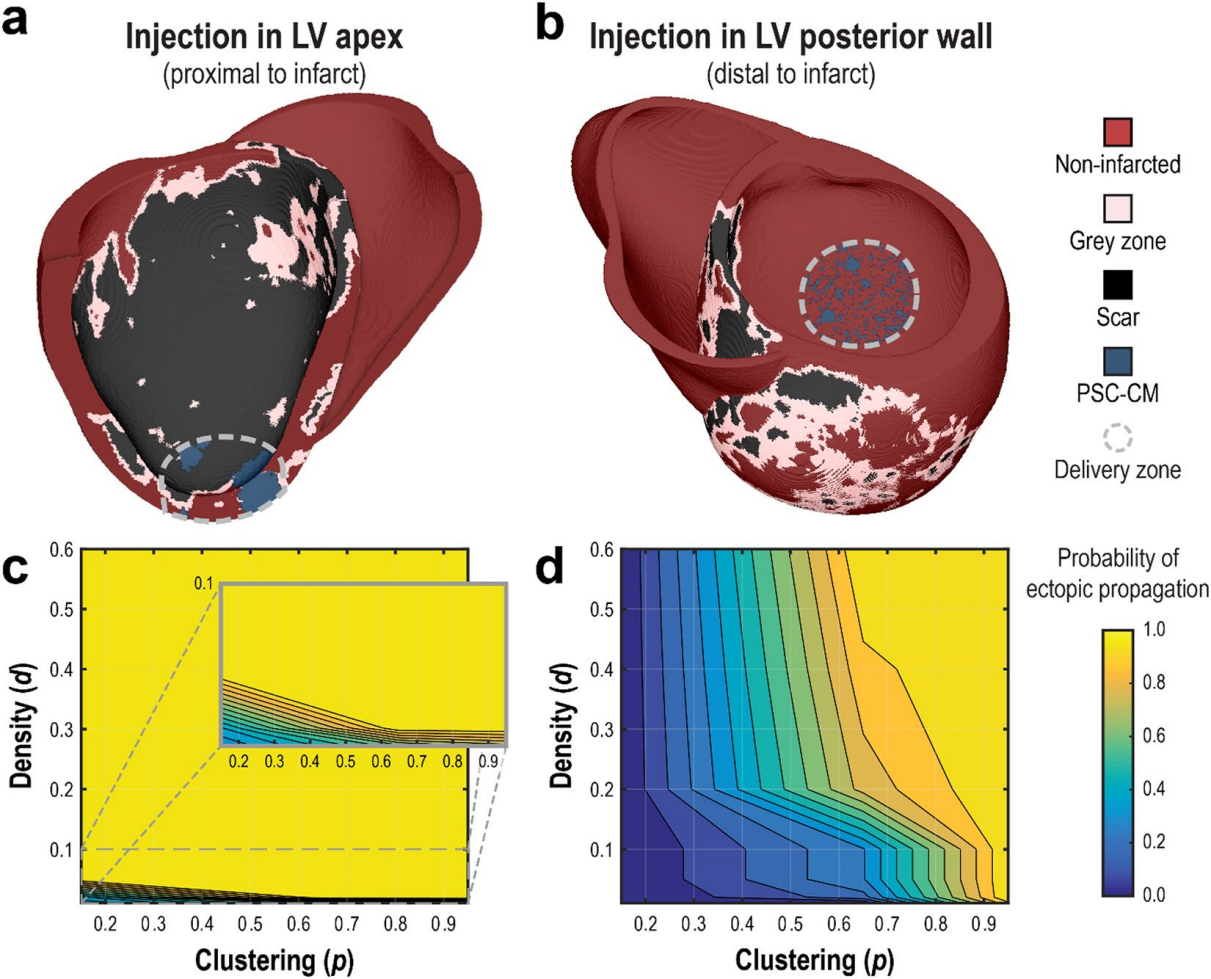
Ectopic propagation arising from PSC-CM automaticity following intramyocardial injection cell delivery is sensitive to spatial clustering (tissue scale) of engrafted PSC-CMs and delivery site location (organ scale). Cell delivery via intramyocardial injection into (**a**) the LV apex and (**b**) and the LV posterior wall was simulated. Representative spatial distributions of simulated PSC-CM engraftment ((**a**) *d* = 0.40, *p* = 0.95, (**b**) *d* = 0.60, *p* = 0.65) are shown for a delivery zone with radius = 1.6 cm. (c,d) The probability of ectopic propagation is plotted as a function of *d* and *p* for delivery into (**c**) the LV apex and (**d**) LV posterior wall.

For heart repair with *de novo* CMs, issues of optimal therapy dose and site of localized delivery are explored at the organ scale. Where and in what amounts *de novo* CMs should be delivered into the post-MI ventricles (Fig. 4a) to facilitate functional recovery while also preventing arrhythmogenesis remains unknown. In the context of cell delivery with engineered cell sheets, cell dosage can be adjusted by altering sheet size and thickness (Fig. 4b). Because PSC-CMs in engineered cell sheets can be aligned to give rise to physiological electrical conduction anisotropy^73^, the simulation platform is capable of exploring the impact of different transplanted cell sheet orientations relative to the local myofiber orientations (Fig. 5c), and whether it results in arrhythmogenicity.

Having described the components of our simulation framework, we next demonstrate how it can be used to gain mechanistic insights into potential arrhythmogenic mechanisms arising from cell therapy. Examples of such insight are provided in the following sections.

### *Arrhythmogenic Mechanism*: Ectopic propagations triggered by PSC-CM automaticity following cell delivery via intramyocardial injection is dependent on injection location, cell dosage, and engraftment spatial distribution

In the first illustrative example, we examined how parameters in cell delivery via intramyocardial injection could lead to ectopic propagations arising from the intrinsic automaticity of PSC-CMs, and under what circumstances. Specifically, we examined the role of engraftment spatial distribution (tissue scale), injection location (organ scale), and cell dosage (organ scale). Injection of PSC-CMs was simulated into a biophysically-detailed, patient-specific model of the human post-MI ventricles; the heart model was previously constructed and used in a study of VT risk stratification by our group^48^. PSC-CM injection was simulated at two sites: the left ventricular (LV) apex adjacent to the infarct (Fig. 5a), and the LV posterior wall distal to the infarct (Fig. 5b). Within the delivery zone (grey dashed line, r = 1.6 cm), or the region around the injection site where PSC-CMs engraft, stochastic spatial distributions of engrafted PSC-CMs were generated across a range of *d* (0.01, 0.02, 0.05, 0.10, 0.40, 0.60) and *p* (0.15, 0.65, 0.95) values (n = 10 for each combination of *d* and *p*). Tissue conductivity within regions of engrafted PSC-CMs was isotropic and reduced (1% of ventricular CM in the orthogonal fiber direction) because gap junction expression is minimal and distributed circumferentially in PSC-CMs during the initial stages of engraftment^59,60^. Simulations for each model configuration were then observed for the presence of spontaneous ectopic propagation originating from locations in the delivery zone; subsequently, the probability for ectopic propagation, or the fraction of simulations observed with focal ectopy, was recorded for each combination of *d* and *p* (n = 10).

Results for simulated injection adjacent and distal to the infarct are summarized in Fig. 5c,d, respectively. Cell delivery bordering the infarct almost always resulted in ectopic propagations, regardless of spatial distribution of engrafted PSC-CMs. In contrast, delivery distal to the infarct into non-infarcted myocardium resulted in significantly fewer ectopic propagations. Specifically, no ectopic propagations were observed for the patchiest spatial distributions (*p* = 0.15) regardless of density (*d*). For moderate degrees of patchiness (*p* = 0.65), incidences of ectopic propagation rose monotonically with increasing cell dose (i.e., increasing values of *d*); ectopic beats were almost always observed for high degree of clustering (*p* = 0.95) regardless of cell dose.

Overall, our model predicts that the propensity for ectopic propagation triggered by PSC-CM automaticity rises with higher cell dose (i.e., higher values of *d*), increased cell clustering (i.e., higher values of *p*), and delivery along the boundary of the infarct. For an ectopic propagation to occur, engrafted PSC-CMs (current source) must generate sufficient depolarizing current to bring the adjacent, repolarized host myocardium (current sink) to its activation threshold. While cell dose alters the total amount of depolarizing current arising from PSC-CM automaticity, cell clustering changes the local depolarizing current density. In contrast, the local tissue composition of the delivery site determines the strength of the current sink. Compared to non-infarcted myocardium, grey zone is a weaker electrical sink due to ionic (i.e., less IK1 and a higher resting membrane potential) and gap junction (i.e., poorly coupled) remodeling. Scar is not an electrical sink at all because it is unexcitable and nonconducting. Consequently, delivery along the boundary of an infarct resulted in significantly more ectopic propagations triggered by PSC-CM automaticity because of a substantially weaker electrical sink. Overall, these simulations provide an example of how one can explore optimal intramyocardial cell delivery based on personalized heart geometry and distribution of scar.

### *Arrhythmogenic Mechanism*: Bundle branch block arising from iCM engraftment onto Purkinje fibers

In the second illustrative example, we sought to determine the electrophysiological ramifications of iCM engraftment onto the Purkinje system, a potential collateral complication of transdifferentiation. We used a biophysically-detailed rabbit ventricular model with representation of Purkinje fibers previously used by the Vigmond group to study arrhythmia mechanisms of the Purkinje system^71,74,75^. The choice of model highlights the versatility of our modeling platform in addressing mechanistic questions and aiding *in vivo* investigations exploring arrhythmia mechanisms of cell therapy across different animal species. We hypothesized that iCM engraftment onto the Purkinje system could give rise to conduction block; the hypothesis is based on our observations of action potential prolongation in ventricular CMs, as illustrated in Fig. 2d.

**Figure 6:**
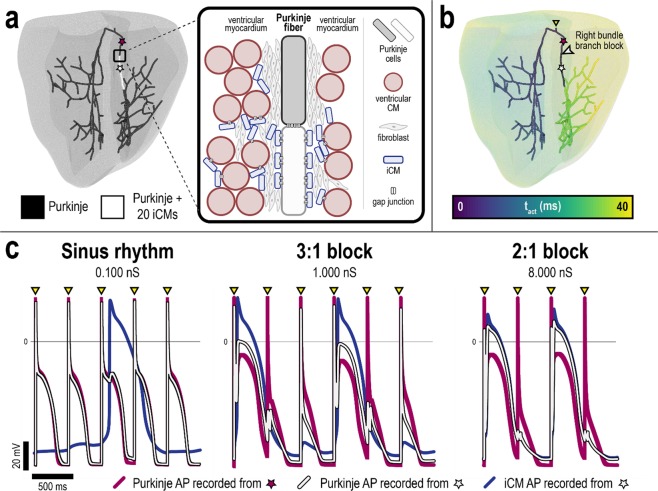
Arrhythmogenic consequences of iCM engraftment onto the Purkinje system following transdifferentiation in a rabbit ventricular model. (**a**) Purkinje system with iCM engraftment onto a contiguous segment of the right bundle branch (RBB, white); 20 iCMs were coupled to a single Purkinje cell along the affected segment. Illustration of how collateral iCM engraftment onto the Purkinje could arise from transdifferentiation (inset). (**b**) RBB block occurred superior to the site of iCM engraftment when iCM-Purkinje junctional conductance was greater than 0.100 nS. Colors indicate time sequence of activation (ms) relative to simulated sinus beat. (**c**) Action potential traces obtained from RBB sites indicated in (**a**,**b**) for different values of iCM-Purkinje junctional conductance. All paced beats propagated through the RBB at 0.100 nS (left); 3:1 block and 2:1 block in the RBB was observed at junctional conductances of 1.000 (middle) and 8.000 nS (right), respectively.

Engraftment along a contiguous segment of the right bundle branch (RBB) of the Purkinje system (0.605 cm) was simulated to model localized transdifferentiation in the superior ventricular septum (Fig. 6a). A single Purkinje cell was coupled to 20 iCMs along the specified RBB segment; such a high number of coupled iCMs could feasibly arise from a combination of cell size mismatch, high transfection efficiency, or local clonal expansion of iCMs. A range of junctional conductance values (0.1 to 8.0 ns) was implemented to represent different levels of electrotonic coupling between iCMs and Purkinje cells. We simulated sinus rhythm in the rabbit heart by pacing from the bundle of His at a basic cycle length of 400 ms. Additional details can be found in the Methods section.

Results revealed that both normal excitation of the Purkinje system as well as RBB block superior to the site of iCM engraftment, as shown in Fig. 6b, can take place, depending on the level of electrotonic coupling between iCMs and Purkinje cells. At 0.100 nS, all paced beats propagated through the RBB (Fig. 6c, left). Poor coupling between iCMs and Purkinje cells in the affected RBB segment severely limited electrotonic interaction between the two; Purkinje cell depolarizations did not result in iCM depolarizations and iCMs were observed to spontaneously beat independent of Purkinje cells. However, 3:1 RBB block and 2:1 RBB block were observed at 1.000 nS and 8.000 nS, respectively (Fig. 6c, middle and right). This arose in part because increased electrotonic coupling between iCMs and Purkinje cells alters Purkinje cell excitability and repolarization. At 1.000 nS, junctional current from depolarizing Purkinje cells was sufficient to elicit iCM depolarization but only on every third sinus beat. During repolarization of the first sinus beat, electrotonic current flow from iCMs to Purkinje cells significantly prolonged the Purkinje action potential; this rendered Purkinje cells in the affected RBB segment refractory and unexcitable for the subsequent sinus beat. Following the blocked sinus beat, Purkinje cell depolarization did not elicit iCM depolarization; the reason for this is that junctional current flow from Purkinje cells was insufficient to excite iCMs still refractory from the previous beat. At 8.000 nS, this was not the case; junctional current flow from Purkinje cells was sufficient to excite refractory iCMs, giving rise to 2:1 RBB block. Overall, these findings demonstrate that collateral iCM engraftment onto the Purkinje system can give rise to severe cardiac conduction abnormalities, an important consideration for clinical safety. The simulation platform can be further employed to explore the severity and dynamics of conduction abnormalities arising from different locations and different spatial distributions of iCM engraftment.

### *Arrhythmogenic Mechanism*: The transplanted PSC-CM cell sheet as a substrate for macroscopic reentrant VT

**Figure 7:**
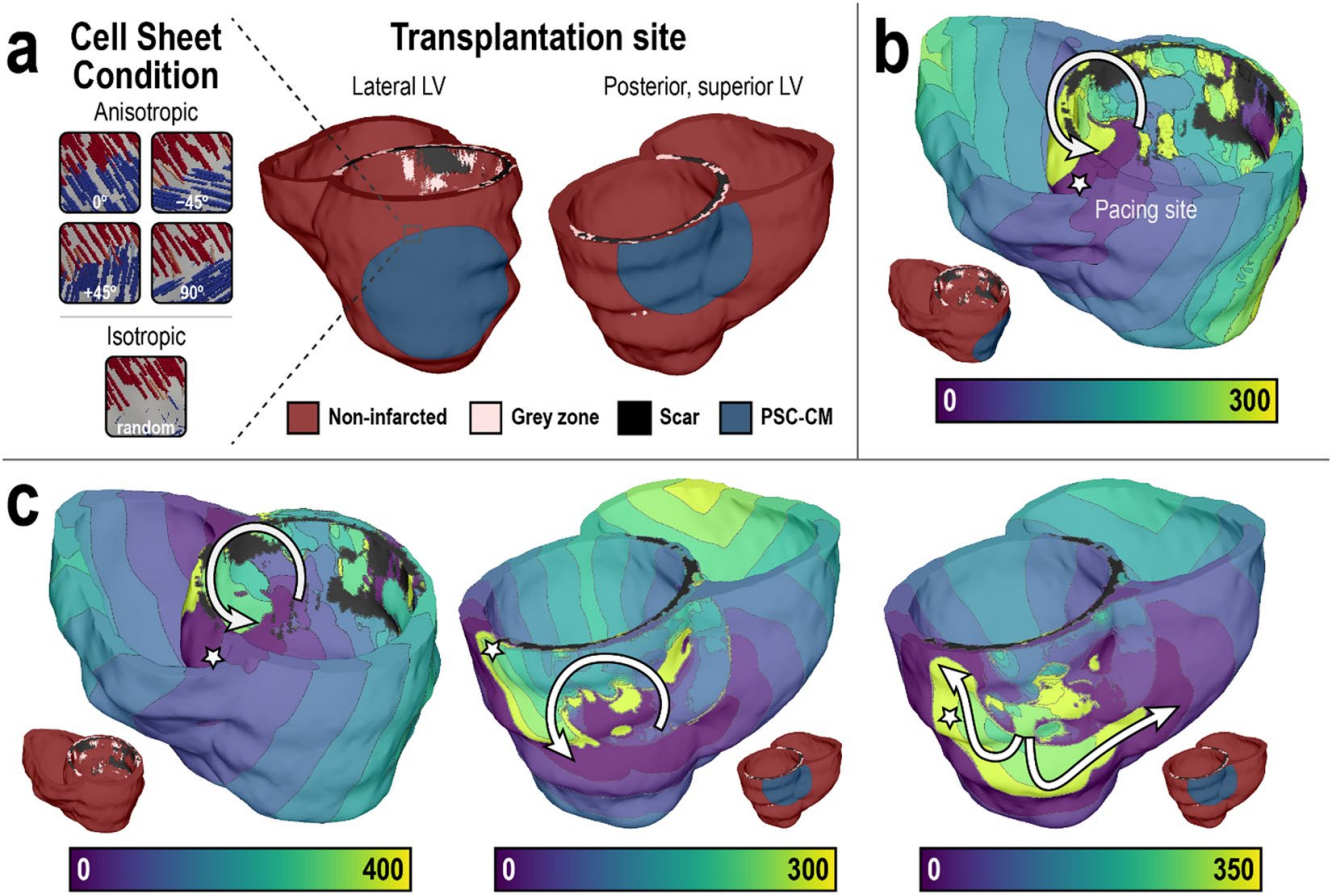
Transplantation of PSC-CM cell sheets results in reentrant VT in a 3D personalized heart model of ischemic cardiomyopathy. (**a**) Cell sheet transplantation (radius, r = 3.2 cm) was simulated at two sites: the lateral LV (middle) and posterior, superior LV (right). Five different cell sheet conditions (anisotropic [0°, ±45°, 90°] isotropic) were simulated (left). (**b**,**c**) Induced reentrant arrhythmias in the treated post-MI heart obtained following pacing from the site indicated by the star. Geometric models are presented together with electrical activation isochronal maps. Colors indicate time sequence of activation (ms). White arrows represent the direction of propagation of reentrant arrhythmias. (**b**) Reentrant VT morphology following cell sheet transplantation onto the lateral LV epicardium. (**c**) Reentrant VT morphologies following cell sheet transplantation onto the posterior, superior LV epicardium.

In the last illustrative example, we used the simulation platform to compare the likelihood of induction for reentrant VT in the post-MI ventricles prior to and after simulated transplantation of PSC-CM cell sheets. PSC-CM cell sheet transplantation was simulated in another patient-specific, MRI-based human model of the post-MI ventricles; the heart model was also previously constructed and used in a study of VT risk stratification by our group^48^. Transplantation and engraftment of PSC-CM cell sheets with clinically relevant dimensions (diameter = 3.2 cm)^76^ was simulated onto the epicardium of the posterior LV directly over the infarct site (Fig. 7a, right) and onto the epicardium of the lateral LV free wall, straddling normal and infarcted myocardium (Fig. 7a, left). The rationale for the latter transplantation site was that improved blood flow, such as that arising from engraftment onto non-infarcted myocardium, has been shown to aid in graft survival^77^. Isotropic (i.e., random myofibril orientation) and anisotropic cell sheets was represented to determine the effects of conduction anisotropy in cell sheets arising from the alignment of PSC-CMs; in the latter condition, four different transplantation orientations were simulated: 0°, ± 45°, and 90° relative to the local fiber direction. VT inducibility was examined in all models, pre- and post-treatment, using our validated VARP protocol^48^, where the ventricles were paced rapidly from 19 uniformly distributed sites to induce VT and examine VT morphologies. The arrhythmogenic propensity of the substrate was assessed by the number of pacing sites from which VT was induced and the number of the different VT morphologies.

Before treatment, reentrant VT was not inducible in the post-MI heart model; this is consistent with the patient’s clinical outcome, who did not have a discharge of the implanted defibrillator device over the follow-up period^48^. Simulated cell sheet transplantation onto the lateral LV free wall in the patient heart model resulted in VT induction. In the isotropic cell sheet case, only one out of the 19 pacing sites induced VT; the resulting reentrant wave was stationary around the superior posterior LV near the RV insertion point (Fig. 7b). The same result was also observed with anisotropic cell sheets for all transplantation orientations. Similarly, simulated cell sheet transplantation onto the posterior LV also resulted in VT induction. Observed VT morphologies are shown in Fig. 7c. In the isotropic cell sheet case, two of the 19 pacing sites induced VT. The VTs had the same morphology; reentrant waves were stationary around the superior posterior LV near the RV insertion point (Fig. 7c, left). In the anisotropic cell sheet case transplanted at 0° and ± 45° orientations, two pacing sites also induced VT. However, the VTs had distinct morphologies. In one, reentrant waves were stationary around the superior, posterior LV (Fig. 7c, middle); in the other, reentrant waves were stationary around the posterior LV free wall (Fig. 7c, right). In the anisotropic cell sheet case transplanted at 90° orientation, only a single pacing site induced VT; reentrant waves were stationary around the superior posterior LV (Fig. 7c, middle). Lastly, no ectopic propagations triggered by PSC-CM automaticity from the cell sheets was observed.

In this example, treatment with PSC-CM cell sheet of clinically relevant dimensions was arrhythmogenic; simulated transplantation and engraftment of PSC-CM cell sheets created an arrhythmogenic substrate and the ventricles became inducible for reentrant VT, while the original heart model was not, despite the presence of an infarct. Transplantation onto the posterior LV directly over the infarct was more arrhythmogenic compared to transplantation onto the lateral LV along the border of the infarct. This finding is explained by the additional substrate heterogeneity arising from PSC-CM cell sheet transplantation; compared to transplantation onto the lateral LV, transplantation onto the posterior LV directly over the infarct exacerbated the electrophysiological heterogeneity, creating a substrate more conducive to reentry^78^. While myofiber anisotropy and transplantation orientation did not alter VT inducibility when the PSC-CM cell sheet was transplanted onto the lateral LV, myofiber anisotropy and transplantation orientation altered the inducibility when the PSC-CM cell sheet was transplanted onto the posterior LV. This finding is explained by the modulation of conduction velocity by myofibril orientation; however, conduction velocity slowing arising from myofibril orientation was only sufficient to affect VT inducibility when PSC-CM cell sheet was transplanted onto the posterior LV. Lastly, reentrant waves were stationary around the posterior LV in heterogeneous regions of scar and GZ in all cases of induced reentrant VTs. This arises from conduction slowing as a result of PSC-CM cell sheet engraftment; conduction slowing, even in regions remote to the infarct when transplantation occurred onto the lateral LV, enabled the development and sustaining of reentry in the infarcted heart^49^. Taken altogether, transplantation location and its effect on substrate heterogeneity appears to be a primary determinant of VT inducibility, while myofiber anisotropy and transplantation orientation and their effect on conduction velocity slowing act as secondary determinants.

## Discussion

In this study, we developed a comprehensive framework for multiscale cardiac electrophysiology simulations of cell-based heart repair with *de novo* CMs. Realistic features of therapy were represented at each model scale. At the cell scale, we developed a methodology for representing two primary treatment modalities: cell delivery of exogenous PSC-CMs and transdifferentiation of endogenous non-myocytes into iCMs. At the tissue scale, we represented heterogeneous spatial distributions of remuscularized regions arising from cell delivery or transdifferentiation. At the organ scale, the framework was designed to simulate different clinical delivery strategies into a 3D heart model and examine its impact on arrhythmogenesis.

The utility of the framework was demonstrated in three case studies exploring the mechanisms of post-therapy arrhythmias. First, we showed that PSC-CM dosage, spatial distribution of engraftment, and injection location were critical determinants of ectopic propagations arising from intrinsic PSC-CM automaticity following cell delivery via intramyocardial injection. Second, we demonstrated that collateral iCM engraftment onto Purkinje fibers following transdifferentiation could lead to conduction block and thus heart rhythm dysfunction. Finally, we demonstrated that transplantation of PSC-CM cell sheets in a human, post-MI heart can render it prone to developing reentrant VT. Highlighting the versatility of our modeling framework to aid in both *in vivo* and clinical studies, simulations were carried out in three different biophysically-detailed 3D heart models: two of which were human, patient-specific models of post-MI and one of which was a rabbit heart with representation of the Purkinje system. These simulations demonstrate how computational modeling can contribute towards the broader technological effort to develop arrhythmia-free, cell-based therapies for cardiac remuscularization. Towards this goal, potential mechanisms of arrhythmogenesis need to be identified, the role of contributing factors need to be explored, and novel strategies need to be devised.

Focal VT arising from ectopic beats triggered by PSC-CMs have remained a constant concern because PSC-CMs spontaneously beat and have been observed to be prone to early and delayed afterdepolarizations^79^. Recently, Liu *et al*.^80^ provided evidence for focal ectopy arising from PSC-CM automaticity as the mechanistic basis for acute VT observed in post-MI macaques injected with human embryonic stem cell-derived cardiomyocytes (hESC-CMs). Electrophysiology studies of hESC-CM treated hearts at 2 weeks following cell injection revealed ectopic propagations originating from a region of the ventricles where hESC-CMs were delivered. Additionally, overdrive pacing of hESC-CM-treated hearts could not terminate the VT. In our case study, we demonstrated that the presence of ectopic propagations triggered by PSC-CM automaticity was determined by source-sink effects. Our simulation framework provides an ideal platform for further analysis of this problem and determining critical safety thresholds prior to human clinical trials. A variety of strategies could be implemented to minimize ectopic propagations arising from PSC-CM automaticity. One approach that could be explored are methods that decrease the intrinsic rate of PSC-CM automaticity; this would enable the overdrive suppression of PSC-CM pacemaker activity. Outside of enhancing PSC-CM maturation, the rate of PSC-CM automaticity can be decreased by enhancing I-K1 current in PSC-CMs via viral transfection^81^ or CRISPR-CAS9^82^. Alternatively, methods that enhance source-sink mismatch could be explored. PSC-CMs could be co-delivered with fibroblasts that act as passive current sinks^83^. The patchiness of engrafted PSC-CMs could be increased by altering the viscosity of the cell suspension solution to maximize PSC-CM dispersion upon injection. Poor electrotonic coupling of engrafted PSC-CMs could be potentially improved by enhancing connexin expression.

Because of poor electrotonic coupling, reentrant VT arising from conduction slowing is also a significant concern. Additional conduction slowing can also be attributed to the isotropic distribution of connexin-43^22,23^. Slowed conduction has been observed in engraft regions of PSC-CMs following cell delivery via intramyocardial injections^22,23^ and in PSC-CM cell sheets *in vitro*^73,84^. In addition to slowed conduction, PSC-CMs can also exhibit significant electrophysiological heterogeneity because they are comprised of a variety of cardiac subtypes (i.e., nodal-, atrial-, and ventricular-like)^85–87^; however, different enrichment^88–90^ and purification^91^ techniques can be used to obtain a ventricular-like population. In confluent preparations of PSC-CMs *in vitro*, conduction velocity and action potential variability has been observed to persist^44,85^. The combination of conduction slowing, variable conduction, and electrophysiological heterogeneity in regions of engrafted PSC-CMs can create a vulnerable substrate for reentrant VT. Despite this, recent *in vivo* results suggest that cell delivery either via intramyocardial injection or transplantation of cell sheets does not alter inducibility for reentrant VT; programmed electrical stimulation was used to test the vulnerability of post-MI hearts for reentrant VT. In cell delivery via intramyocardial injection, Liu *et al*. determined that there was no significant difference in the arrhythmia inducibility of post-MI macaque hearts between hESC-CM-injected and control groups at two weeks following treatment^80^. In cell delivery via PSC-CM cell sheets, Ling Gao *et al*.^76^ did not observe any significant increase in arrhythmias when PSC-CM cell sheets of a similar size were transplanted onto post-MI porcine hearts. Incidences of spontaneous ventricular arrhythmias in the PSC-CM cell sheet-treated group were not significantly different compared to the two control groups (post-MI porcine hearts without treatment or post-MI porcine hearts treated with an acellular fibrin patch). Programmed electrical stimulation at 4 weeks post treatment confirmed this result. While these experimental results differ from our simulation results, numerous factors could have contributed. In Liu *et al*.’s study, the smaller size of the macaque heart compared to that of a human could have limited the inducibility for reentrant VT following programmed electrical stimulation. In Ling Gao *et al*.’s study, cell sheets had only a ~10% rate of engraftment. Our framework enables further analysis of the role of these discrepancies on inducibility for reentrant VT.

While collateral engraftment of PSC-CMs or iCMs onto the Purkinje has not been experimentally reported, should it be proven to occur, it could lead to conduction block as we demonstrate in this study. Furthermore, if PSC-CMs or iCMs engrafted onto the Purkinje were to also engraft onto adjacent myocardium, abnormal ventricular excitations could also arise. Future work could analyze the severity and persistence of conduction abnormalities arising from collateral iCM or PSC-CM engraftment in a human heart model and explore how this could interact with ectopic propagations to give rise to a vulnerable substrate for reentry that includes the Purkinje system.

Significant intersubject variability in arrhythmia type and prevalence has been observed *in vivo* in studies of cell delivery into post-MI macaque hearts^21,24,80^ making drawing conclusions about clinical safety extremely difficult. It remains unknown what factors give rise to one type of arrhythmia in one animal, another type of arrhythmia in another, and none in others. This could be due in part to many possible arrhythmia mechanisms, as we show; this could also be due in part to patient-specific considerations. In a different patient-specific post-MI model (data not shown), we observed that transplantation and engraftment of PSC-CM cell sheets could surprisingly reduce VT inducibility. The temporal dynamics of cell engraftment and electrotonic coupling is yet another contributing factor, especially in the days and weeks following therapy. *In vivo*, the prevalence of ventricular arrhythmias was observed to be the highest around two weeks following cell delivery before declining in subsequent weeks^21,24^. In our second case study, we demonstrated that bundle branch block was affected by the degree of electrotonic coupling of collaterally engrafting iCMs. In future work, we plan to determine and further analyze the contribution of graft coupling on the severity and persistence of ventricular arrhythmias presented here. Taken altogether, these findings suggest that treatment needs to be tailored to the specific individual–with numerous variables optimized and different temporal scales considered. Our simulation framework provides an ideal platform to further explore how this can be accomplished by first broadly understanding the mechanisms and parameters driving arrhythmogenesis and then providing a strategy for individualized treatment planning. To better understand the broader mechanism however, robust methods of *in vivo* cell tracking^92^ need to be implemented that enable the generation of accurate, geometric 3D heart models across time.

Although our framework integrates all presently available information regarding cell-based heart repair with *de novo* CMs, there are still numerous unknown physiological factors. However, the modularity of our simulation framework enables easy integration of new experimental findings at the appropriate scales of hierarchy. PSC-CMs and iCMs have been shown to morphologically and genetically mature *in vivo*^24,36,59,60^; however, a detailed electrophysiological characterization of the changes that occur during maturation has not been achieved, as the exact mechanisms remain unknown. This maturation has been observed to initially occur along the periphery of engrafted sites along the interface with viable host myocardium; it is believed that this maturation might be due in part to enhanced mechanical stimulation of the PSC-CMs located adjacent to actively contracting myocardium. Proper mechanical stimulation (i.e., substrate stiffness^93^ and cyclic mechanical stretching^94,95^) has been shown to be an important driver for maturation of PSC-CMs *in vitro*. Other aspects of the local tissue environment, specifically the degree of ischemia and inflammation, has also been shown to affect cell viability and engraftment^96^. Compared to uninjured hearts, regions of engrafted PSC-CMs in the post-MI guinea pig heart exhibited more variation in graft size, spatial distribution, and electrotonic coupling^23^. Furthermore, chronic or acute inflammation could alter the subsequent fibrotic remodeling of the electrophysiological substrate, but how that occurs and whether the two processes differ remains unclear. In the context of remuscularization, acute inflammation can arise from post-MI healing itself or from injury arising from intramyocardial injection; transplantation of allogeneic PSC-CMs could lead to chronic inflammation. Additionally, PSC-CMs have also been shown to secrete exosomes that exert paracrine effects on native ventricular CMs as well^4,97^. However, the paracrine effects of secreted exosomes on the membrane kinetics of peri-infarcted border zone ventricular cardiomyocytes remain poorly characterized as well as their release and transport kinetics *in vivo*. These inherent uncertainties reaffirm the importance of our framework’s modularity—as new details become available, they can be integrated at the appropriate levels of model hierarchy and new and more accurate model results can be obtained.

As the framework continues to be refined and expanded, it can be used to explore additional exciting developments and applications of cell therapy. One avenue of exploration would be the use of PSC-CMs as biological pacemakers by leveraging their intrinsic automaticity; our simulation framework could aid in the exploration of source-sink effects to ensure robust and stable pacing from sinoatrial-like PSC-CMs^98^. Another avenue of exploration is the integration of a coupled electromechanical model of the ventricles. With the development and integration of a PSC-CM or iCM myofilament model, we would be able simulate the degree of contractile improvement arising from remuscularization following cell therapy. A full electromechanically coupled 3D heart model would enable researchers to truly optimize cell delivery to maximize restoration of contractility and minimize risk of arrhythmia.

Our computational framework for simulating cell-based heart repair with *de novo* CMs is a predictive tool that will expedite clinical translation by driving hypotheses development and providing a platform for detailed mechanistic enquiry at the preclinical stage. Specifically, our framework can provide insight and guidance that could help anticipate pitfalls and minimize expensive, time-consuming *in vivo* experiments. At the clinical stage, our framework would offer patient-specific treatment planning and risk stratification. The modularity of our simulation hierarchy enables us to apply it to any heart regardless of species, size, and pathological state. Our comprehensive simulation framework represents a significant step forward in cardiac regenerative medicine by applying *in silico* cardiac electrophysiological modeling in the quest to understand the mechanistic basis of unwanted arrhythmias.

## Methods

### Modeling PSC-CM engraftment in cell delivery and iCM engraftment in transdifferentiation

We modeled the membrane kinetics of both PSC-CMs and iCMs via the Paci *et al*. formulation, which is calibrated to match experimental recordings from human induced pluripotent stem cell-derived cardiomyocytes (hiPSC-CMs)^54^. Our rationale for using the same ionic models for both types of cells is that no biophysically-detailed mathematical model of iCMs based on patch-clamp recordings exists; moreover, the exact characteristics and evolution of iCM membrane kinetics *in vivo* remains unknown. However, there are broad *in vitro* morphological and electrophysiological similarities between PSC-CMs and iCMs (i.e., action potential shape and observed cardiac subtypes)^55,56,99^. Between the atrial-like and ventricular-like hiPSC-CM formulations, the ventricular-like subtype was chosen because it is the most appropriate type for remuscularization of the ventricles. The corresponding ionic model was downloaded from the CellML repository (https://www.cellml.org/). Model modifications were incorporated to better represent remuscularized regions arising from transdifferentiation *in vivo*.

While the membrane behavior of remuscularized regions in cell delivery were modeled exclusively by that of PSC-CMs, the membrane behavior of remuscularized regions in transdifferentiation was represented as a coupled host myocyte-iCM tandem. This representation was chosen because remuscularized myocardium in cell delivery has been reported to consist of distinct, contiguous clusters of engrafted PSC-CMs^21,24,80^ while remuscularized myocardium in transdifferentiation consists of engrafted iCMs distributed much more diffusely^36^. The latter arises because transdifferentiation targets fibroblasts in the interstitium between host myocytes. Because simulated cardiac tissue cannot be discretized at the spatial resolution necessary to represent individual cells and remain computationally feasible, we chose instead to model diffusely distributed iCMs using a cell tandem approach^100^. A similar approach has been used by our lab to simulate the delivery and engraftment of light-sensitized HEK cells into the heart^46^. Specifically, at each finite element node in a remuscularized region affected by transdifferentiation, modified cells were represented by resistive coupling of several iCMs to the “host” myocyte, the membrane kinetics of which depended on the tissue type (i.e., non-infarcted or grey zone myocardium, or Purkinje cell). Junctional current flow (*I*_*junctional*_) between iCMs and host myocytes was calculated by the following equation:

$${I}_{{junctional}}=-{g}_{{junctional}}({V}_{m{,}{host}}-{V}_{m{,}{iCM}})$$

where *g*_*junctional*_ is the coupled host myocyte-iCM junctional conductance, *V*_*m*,*host*_ is the host myocyte transmembrane potential, and *V*_*m*,*iCM*_ is the iCM transmembrane potential. By convention, *I*_*junctional*_ is negative if current flow is out of the host myocyte. Because iCMs are much smaller (10 × 15 μm) than mature myocytes (100 × 25 μm)^56,99^, numerous iCMs can engraft onto a single host myocyte. This was factored into the host myocyte membrane model using the equation:

$${I}_{c{,}{host}}={n}_{{iCM}}(\,-\,{I}_{{junctional}})/{C}_{m{,}{host}}+\sum _{i=1}^{n}{i}_{i{,}{host}}$$

where *I*_*c*,*host*_ is the net ionic current across the host myocyte lipid membrane, *n*_*iCM*_ is the number of coupled iCMs, *C*_*m*,*host*_ is the host myocyte membrane capacitance (185 pF), and ${\sum }^{}{i}_{i,host}$ is the sum of the ionic currents (i.e., Na^+^, Ca^2+^, K^+^) of the host myocyte. Both *g*_*junctional*_ and *n*_*iCM*_ were specified in simulations of collateral iCM engraftment onto the Purkinje system in a rabbit model. Junctional conductance, *g*_*junctional*_, was varied between 0.1 to 8.0 ns to represent different levels of electrotonic coupling. A single Purkinje cell was coupled to *n*_*iCM*_ = 20 along the affected Purkinje bundle; we determined that this configuration was reasonable given the immense size mismatch between the two cell types (i.e., Purkinje cells are approximately the size of a ventricular myocyte if not larger) and linear arrangement of Purkinje cells in Purkinje fibers. In organ scale simulations, coupling between host myocytes remained unchanged in regions with engrafted iCMs. We assumed this because gap junction remodeling has not been shown to occur following transdifferentiation^36^.

### Simulated spatial distribution of *de novo* CMs

To simulate the spatial distribution of remuscularized regions of engrafted *de novo* CMs (i.e., PSC-CM in cell delivery or a host myocyte:iCM tandem in transdifferentiation), we implemented a stochastic algorithm previously used to model fibrosis distribution^61^ and the distribution of ChR2-expressing cells in models of light-sensitized hearts^46^. The physiological basis for these spatial patterns around sites of intramyocardial injection is a combination of cell death, cell migration, and cell proliferation. First, a 3D delivery zone denoting the localized site where *de novo* CMs could potentially engraft was prescribed by a sphere of radius (r) centered around a given endocardial node of the finite element mesh. Subsequently, stochastic spatial distributions of engrafted *de novo* CMs within this delivery zone were generated based on the parameters *d* and *p*; these parameters modulated the density or cell dosage (by volume fraction of delivery zone) and degree of spatial clusteredness, respectively. From a list of the *N* contained elements within the delivery zone, elements were successively tagged remuscularized until the number of tagged elements was ≥ d times *N*. For each new element tagged remuscularized, the probability of adding to an existing remuscularized cluster or starting a new cluster was given by *p*.

### Clinical imaging-based human heart models

Patient-specific 3D human heart models were previously generated and used in a study of VT risk stratification of post-MI patients by our group^48,101^. Full details of the 3D model reconstruction process and electrophysiological modeling can be found in this previous publication. Below, we briefly summarize this methodology. Finite-element ventricular meshes were derived from clinical LGE-MRI images. For each heart, boundaries of the myocardial wall in the MRI stack were first contoured; within the myocardial wall, regions were classified as non-infarcted, grey zone, and scar based on signal thresholding^48,70^. Geometries of the ventricular wall and of the 3 myocardial regions were subsequently merged using a previously validated methodology based on the variational implicit functions interpolation^66^ to create the final geometric reconstruction. In the reconstructed patient-specific ventricles, fiber orientations were assigned on a per-element basis using a previously validated rule-based approach^102^. Human ventricular cardiomyocyte membrane kinetics in non-infarcted myocardium were simulated using the ten Tusscher-Panfilov formulation^103^. Modifications to the ionic model were incorporated to represent electrophysiological remodeling in grey zone myocardium; specifically, peak sodium current was reduced to 38%, peak L-type calcium current was reduced to 38%, and peak potassium currents I_Kr_ and I_Ks_ were reduced to 30% and 20%, respectively. Infarcted myocardium was modeled as nonconducting and unexcitable. Tissue conductivities in non-infarcted and grey zone myocardium were assigned as previously described^48^ with appropriate adjustments incorporated into the latter to reflect connexin-43 remodeling observed experimentally. Parameter values for each 3D human heart model are summarized in the Supplementary Tables S1 and S2, respectively.

### VT induction protocol

The VARP (Virtual-heart Arrhythmia Risk Predictor) protocol for simulated programmed electrical stimulation was used to determine reentrant VT inducibility; the procedure is described in full detail in our previously published study^48^. Briefly, the ventricular model was subjected to simulated pacing from 19 uniformly distributed endocardial sites (2 in the right ventricle, 17 in the left ventricle). In the left ventricle, pacing locations were automatically assigned^104^ based on the AHA left ventricle segments^105^. This distribution was selected to capture all possible VTs that could arise in the patient-specific, 3D heart model. For each pacing site, rapid pacing was delivered. The pacing train consisted of 8 initial pacing stimuli (S1) at a cycle length of 600 ms followed by a premature stimulus (S2) delivered 300 ms after the last S1 stimulus. If S2 did not result in the generation of arrhythmia, the S1–S2 interval was shortened, in 10 ms steps, until arrhythmia was induced or the S2 failed to capture in tissue. This type of pacing train mimicked ones delivered in standard clinical practice^48,101^. If arrhythmia was still not induced an additional S3, and if necessary S4, was delivered in the same fashion as S2. If arrhythmias did not self-terminate over a 2 second post-pacing period, reentrant VT was induced.

### Simulated collateral engraftment of iCMs onto the Purkinje system in a rabbit heart model

In a biophysically-detailed rabbit ventricular model including a representation of the Purkinje system^71,75^, collateral iCM engraftment onto the Purkinje system was simulated along a contiguous segment (0.605 cm) of the RBB, just distal to the bifurcation at the end of the His bundle. This could arise if transdifferentiation were to target the superior ventricular septum. The rabbit model was previously validated and used in numerous studies of Purkinje-mediated ventricular excitation^71,75^ and arrhythmia^106^. Along the affected RBB, 20 iCMs were coupled to a single Purkinje cell; this could arise due to the immense size mismatch between iCMs (10 × 15 μm) and Purkinje cells (~100 × 25 μm), enabling numerous iCMs to engraft onto a single Purkinje cell especially early on when iCMs are the smaller in size^56,99^. In the rabbit model, ventricular cardiomyocyte and Purkinje cell membrane kinetics were represented by the Mahajan-Shiferaw^107^ and Aslanidi-Sleiman formulations^108^, respectively. Additional 3D model parameters for the rabbit electrophysiological model can be found in the Supplementary Table S3.

### Simulated transplantation and engraftment of PSC-CM cell sheets

A one-element thick layer along the surface of the epicardium around a specified node was selected to simulate transplantation and engraftment of PSC-CM cell sheets. To accomplish this, the subset of points along the epicardial surface of the finite element mesh was first identified. Next, the additional subset of points around a user specified node was determined. Finally, all elements with a vertex that included at least one of those points were identified, thereby specifying the final epicardial region of PSC-CM cell sheet engraftment. Within the cell sheet, PSC-CM electrophysiological properties were assigned to the nodes. 10% tissue conductivity of normal myocardium was assigned to the elements because PSC-CM cell sheets cultured *in vitro* have noticeably slower conduction velocity compared to native ventricular myocardium (~3-fold less)^73^.

Within PSC-CM cell sheet elements, the original fiber orientation of the post-MI heart model was modified to simulate different treatment conditions. In the case of anisotropic cell sheets, different transplantation orientations were simulated by rotating fibers ±45° or ±90° about the surface normal of the nearest epicardial element face. In the case of isotropic sheets, fibers were randomly rotated about the surface normal of the nearest epicardial element face; angles of rotation were randomly sampled from a uniform distribution between ±90°. Complete engraftment was assumed (i.e., conduction between the cell sheet and host myocardium was not different from conduction across the cell sheet).

### Simulation details

Monodomain simulations of electrical activity in the heart models were run using the CARP software package^109,110^ on a parallel computing system. Using the finite-element method, a reaction-diffusion partial differential equation, describing the spread of electrical current through the myocardium, was solved together with the ordinary and algebraic equations, describing myocyte membrane kinetics, at each node of the mesh. Details about computational resources for each set of simulations can be found in Supplementary Tables S1–S3. Simulation results were visualized using Dr. Edward Vigmond’s meshalyzer tool.

### Data and code availability

The software used to generate clinical imaging-based heart models are described in a previous publication^111^. Briefly, patient MRIs are cropped in 3DSlicer (https://www.slicer.org/) and the myocardial walls are segmented using CardioViz3D (http://www-sop.inria.fr/asclepios/software/CardioViz3D/). Simpleware ScanIP, available from Synopsys, is used to generate 3D computational meshes from the segmented images. The set of algorithms and subroutines to assign fiber orientation in the 3D meshes using a rules-based approach are presented in the original publication^102^. Similarly, the set of subroutines used to stochastically assign spatial distribution of *de novo* CMs are presented in the original publication^61^. Patient MRI images and reconstructed models are available upon request and approval of Johns Hopkins Institutional Review Board. The rabbit model with realistic representations of the Purkinje system are available upon request. To set up and run monodomain simulations of electrical activity, ionic models of the human cardiomyocyte, including that of PSC-CM, can be accessed from the CellML repository (https://models.physiomeproject.org/electrophysiology). The electrophysiology simulator CARP software package and accompanying tutorials can be found online (https://carpentry.medunigraz.at/carputils/index.html).

## Supplementary information

Supplemental Information
