# Supplementary material for: A comprehensive, multiscale framework for evaluation of arrhythmias arising from cell therapy in the whole post-myocardial infarcted heart
Source: Sci Rep. 2019 Jun 25;9:9238. doi: 10.1038/s41598-019-45684-0 (PMC6592890; doi:10.1038/s41598-019-45684-0)
Supplement: Supplementary file 1 — Supplemental Information [file 41598_2019_45684_MOESM1_ESM.docx]

**A comprehensive, multiscale framework for evaluation of arrhythmias arising from cell therapy in the whole post-myocardial infarcted heart**

Joseph K. Yu^1^, William Franceschi^1^, Qinwen Huang^1^, Farhad Pashakhanloo^1^, Patrick M. Boyle^1^, Natalia A. Trayanova^1^*

*^1^Institute for Computational Medicine, Johns Hopkins University, 3400 N Charles St, 216 Hackerman Hall, Baltimore, Maryland 21218,* USA

Correspondence and requests for materials should be addressed to Natalia A. Trayanova (ntrayanova@jhu.edu)

# **Supplementary Information**

**Supplementary Table S1:** Simulation details for cell delivery via intramyocardial injection into an MRI-based, 3D heart model of the post-MI, human ventricles

| **Category** | **Parameter(s)** | **Value** |
| --- | --- | --- |
| Geometry | # nodes | 1,495,620 |
|  | # tetrahedral elements | 6,013,582 |
| Conductivity throughout the myocardium (mS/mm) | $\sigma_{iL}$ | 0.255 |
|  | $\sigma_{iT}$ | 0.0775 |
|  | $\sigma_{iN}$ | 0.0775 |
| Conductivity throughout the grey zone (mS/mm) | $\sigma_{iL}$ | 0.255 |
|  | $\sigma_{iT}$ | 0.00775 |
|  | $\sigma_{iN}$ | 0.00775 |
| Computation | # of Intel Xeon E5-2680v3 processors @2.5 GHz | 36 |
|  | Wall time required | 10 hrs |

**Supplementary Table S2:** Simulation details for cell delivery via cell sheets into an MRI-based, 3D heart model of the post-MI, human ventricles

| **Category** | **Parameter(s)** | **Value** |
| --- | --- | --- |
| Geometry | # nodes | 3,306,823 |
|  | # tetrahedral elements | 4,092,396 |
| Conductivity throughout the myocardium (mS/mm) | $\sigma_{iL}$ | 0.1585 |
|  | $\sigma_{iT}$ | 0.0282 |
|  | $\sigma_{iN}$ | 0.0282 |
| Conductivity throughout the grey zone (mS/mm) | $\sigma_{iL}$ | 0.1650 |
|  | $\sigma_{iT}$ | 0.00282 |
|  | $\sigma_{iN}$ | 0.00282 |
| Computation | # of Intel Xeon E5-2680v3 processors @2.5 GHz | 48 |
|  | Wall time required | 18 hrs |

**Supplementary Table S3:** Simulation details for rabbit ventricles and Purkinje system

| **Category** | **Parameter(s)** | **Value** |
| --- | --- | --- |
| Geometry | # nodes | 547,680 |
|  | # Purkinje elements | 1,116 |
| Conductivity throughout the myocardium (mS/mm) | $\sigma_{iL}$ | 0.1845 |
|  | $\sigma_{iT}$ | 0.0239 |
|  | $\sigma_{iN}$ | 0.0239 |
| Purkinje-myocardial junctions | Junctional resistance | 85 MΩ |
|  | Junctional scaling factor | 80,000 |
|  | # ventricular nodes per Purkinje node | 200 to 220 |
|  | Purkinje endpoint penetration depth | 10% |
|  | Purkinje fiber radius | 15 μm |
|  | Purkinje fiber internal conductivity | 0.1 S/m |
|  | Purkinje fiber gap junction resistance | 100 kΩ |
| Computation | # of Intel Xeon E5-2680v3 processors @2.5 GHz | 36 |
|  | Wall time required | 5 hrs |
